# The Practitioner’s Handbook of Treatment; or, the Principles of Therapeutics

**Published:** 1887-08

**Authors:** 


THE PRACTITIONER'S HANDBOOK OF TREATMENT; OR, THE PRINCIPLES OF THERAPEUTICS. By J. Milner Fothergill, M. D., Physician to the City of London Hospital for Diseases of the Chest, Victoria Park; late Assistant Physician to the West London Hospital ; Hon. M. D. Rush College, Chicago, Illinois; Foreign Associate Fellow of the College of Physicians of Philadelphia. Third American from the third English Edition. Philadelphia: Lea Brothers & Company. 1887.

To the philosophic inquirer after truth, the principles involved in the use of remedies must always prove of interest, and the sale of a second and larger edition of this work tells of the favor it finds with its readers. The work has been carefully revised and considerable additions have been made in this third edition. It is confidently recommended as being trustworthy in the elucidation of curative processes that should guide the physician.

